# ExoU Induces Lung Endothelial Cell Damage and Activates Pro-Inflammatory Caspase-1 during *Pseudomonas aeruginosa* Infection

**DOI:** 10.3390/toxins14020152

**Published:** 2022-02-18

**Authors:** Kierra S. Hardy, Amanda N. Tuckey, Phoibe Renema, Mita Patel, Abu-Bakr Al-Mehdi, Domenico Spadafora, Cody A. Schlumpf, Robert A. Barrington, Mikhail F. Alexeyev, Troy Stevens, Jean-Francois Pittet, Brant M. Wagener, Jon D. Simmons, Diego F. Alvarez, Jonathon P. Audia

**Affiliations:** 1 Department of Microbiology and Immunology, College of Medicine, University of South Alabama, Mobile, AL 36688, USA; ksh1004@jagmail.southalabama.edu (K.S.H.); ant1922@jagmail.southalabama.edu (A.N.T.); cas1738@jagmail.southalabama.edu (C.A.S.); rbarrington@southalabama.edu (R.A.B.); 2 Center for Lung Biology, College of Medicine, University of South Alabama, Mobile, AL 36688, USA; prenema@southalabama.edu (P.R.); mitarpatel@southalabama.edu (M.P.); mehdi@southalabama.edu (A.-B.A.-M.); malexeye@southalabama.edu (M.F.A.); tstevens@southalabama.edu (T.S.); jdsimmons@health.southalabama.edu (J.D.S.); diego.alvarez@shsu.edu (D.F.A.); 3 Department of Systems Biology, Harvard Medical School, Boston, MA 02115, USA; 4 Department of Physiology and Cell Biology, College of Medicine, University of South Alabama, Mobile, AL 36688, USA; 5 Department of Biomedical Sciences, College of Allied Health, University of South Alabama Mobile, Mobile, AL 36688, USA; 6 Department of Pharmacology, College of Medicine, University of South Alabama, Mobile, AL 36688, USA; 7 Flow Cytometry Core Lab, College of Medicine, University of South Alabama, Mobile, AL 36688, USA; dspadafora@southalabama.edu; 8 Department of Anesthesiology and Perioperative Medicine, Birmingham School of Medicine, University of Alabama, Birmingham, AL 35294, USA; jpittet@uabmc.edu (J.-F.P.); bwagener@uabmc.edu (B.M.W.); 9 Department of Surgery, College of Medicine, University of South Alabama, Mobile, AL 36688, USA; 10 Department of Physiology and Pharmacology, College of Osteopathic Medicine, Sam Houston State University, Conroe, TX 77304, USA

**Keywords:** *Pseudomonas aeruginosa*, ExoU, pulmonary endothelial cells, caspase-1, mitochondria, pneumonia, sepsis, stress responses, reactive oxygen species

## Abstract

The Gram-negative, opportunistic pathogen *Pseudomonas aeruginosa* utilizes a type III secretion system to inject exoenzyme effectors into a target host cell. Of the four best-studied exoenzymes, ExoU causes rapid cell damage and death. ExoU is a phospholipase A_2_ (PLA_2_) that hydrolyses host cell membranes, and *P. aeruginosa* strains expressing ExoU are associated with poor outcomes in critically ill patients with pneumonia. While the effects of ExoU on lung epithelial and immune cells are well studied, a role for ExoU in disrupting lung endothelial cell function has only recently emerged. Lung endothelial cells maintain a barrier to fluid and protein flux into tissue and airspaces and regulate inflammation. Herein, we describe a pulmonary microvascular endothelial cell (PMVEC) culture infection model to examine the effects of ExoU. Using characterized *P. aeruginosa* strains and primary clinical isolates, we show that strains expressing ExoU disrupt PMVEC barrier function by causing substantial PMVEC damage and lysis, in a PLA_2_-dependent manner. In addition, we show that strains expressing ExoU activate the pro-inflammatory caspase-1, in a PLA_2_-dependent manner. Considering the important roles for mitochondria and oxidative stress in regulating inflammatory responses, we next examined the effects of ExoU on reactive oxygen species production. Infection of PMVECs with *P. aeruginosa* strains expressing ExoU triggered a robust oxidative stress compared to strains expressing other exoenzyme effectors. We also provide evidence that, intriguingly, ExoU PLA_2_ activity was detectable in mitochondria and mitochondria-associated membrane fractions isolated from *P. aeruginosa*-infected PMVECs. Interestingly, ExoU-mediated activation of caspase-1 was partially inhibited by reactive oxygen species scavengers. Together, these data suggest ExoU exerts pleiotropic effects on PMVEC function during *P. aeruginosa* infection that may inhibit endothelial barrier and inflammatory functions.

## 1. Introduction

*Pseudomonas aeruginosa* is an opportunistic, Gram-negative pathogen and a common cause of respiratory infections in mechanically ventilated patients, often leading to acute respiratory distress syndrome (ARDS) and sepsis [1,2,3,4]. *P. aeruginosa* belongs to the ESKAPE group of pathogens (including *Enterococcus faecium*, *Staphylococcus aureus*, *Klebsiella pneumoniae*, *Acinetobacter baumannii, P. aeruginosa*, and *Enterobacter* species) and is currently designated by the World Health Organization as a high priority for development of novel antibiotics [5,6,7,8]. *P. aeruginosa* virulence is primarily driven by the type III secretion system (T3SS) and its cognate exoenzyme effectors, which are associated with poor disease outcomes in patients with pneumonia, ARDS, and/or sepsis [3,4,9]. There are four well-characterized T3SS effectors, each of which is defined by the eukaryotic cofactor(s) required to activate enzymatic function upon injection into a host cell [10,11,12,13]. ExoS and ExoT are bi-functional enzymes with GTPase activating protein and ADP ribosyltransferase activities; ExoY is a nucleotidylyl cyclase; and ExoU is a highly cytotoxic phospholipase A_2_ (PLA_2_) enzyme [9,14,15,16,17,18]. Each of the four exoenzyme effectors differ in the mechanism and/or extent of damaging effects elicited upon injection into a host cell. These differences define the relative contribution of each exoenzyme effector to *P. aeruginosa* virulence.

*P. aeruginosa* clinical isolates vary in the combination of T3SS exoenzyme effectors they encode with a relative distribution of ~35% ExoU, ~65% ExoS, ~100% ExoT, and ~90% ExoY [1]. Importantly, the ExoU PLA_2_ effector protein is associated with some of the highest levels of patient morbidity and mortality [1,2,3,12,19,20,21]. The pathogenic effects of ExoU have been well-described using cell culture and animal infection models (for a recent review, see [22]). ExoU is a highly potent inducer of acute host cell cytotoxicity and death [12,23,24,25,26,27]. ExoU PLA_2_ function is activated primarily by ubiquitin and ubiquitinylated host proteins, and ExoU targets the host plasma membrane to induce rapid cell lysis [14,24,26,28]. In pre-clinical rodent models, inoculation with *P. aeruginosa* strains expressing ExoU results in bacterial colonization of the lungs, increased propensity for dissemination, and lower dose-dependent morbidity and mortality [3,21,25,29,30]. In addition, ExoU disrupts inflammatory signaling and is cytotoxic to epithelial and immune cells [12,23,31,32,33,34,35]. While the effects of ExoU on lung epithelial and immune cells are well studied, a role for ExoU in disrupting lung endothelial cell function has only recently emerged [32,36,37,38,39,40]. Lung endothelial cells are a barrier to proteinaceous fluid flux into tissue and airspaces, and in addition, play an important role in regulating inflammation. In the present study, we sought to examine the interplay between ExoU and the lung endothelial barrier and inflammatory functions.

## 2. Results

### 2.1. P. aeruginosa Strains Expressing ExoU Cause PMVEC Lysis and Barrier Disruption in a Time-, Dose-, and PLA_2_-Dependent Manner

**Figure 1:**
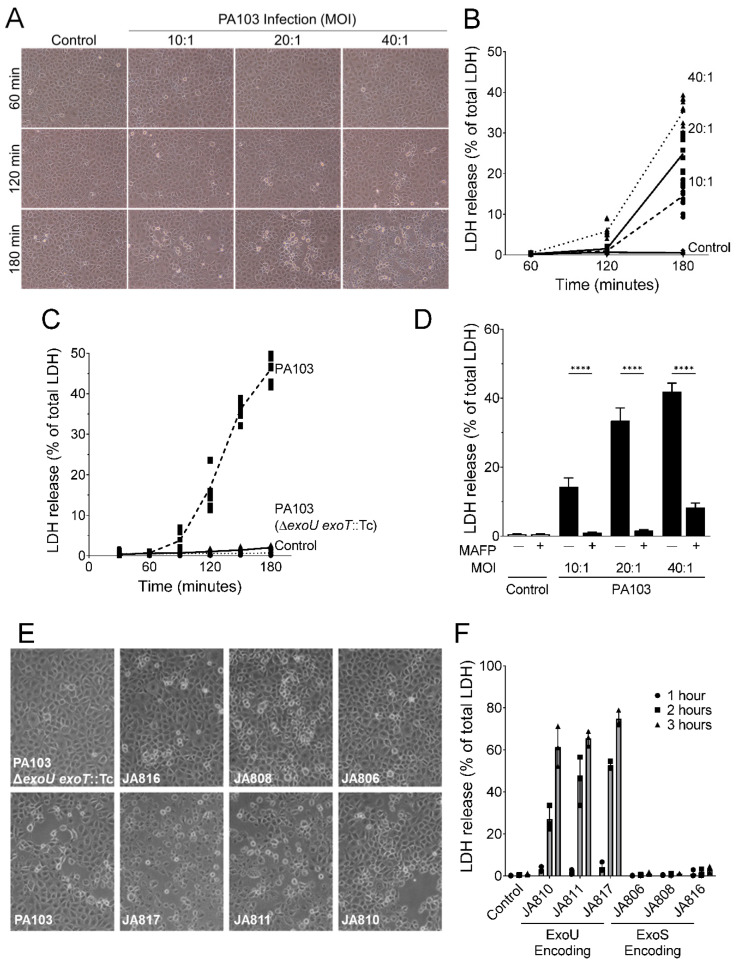
*P. aeruginosa* strains encoding ExoU damage PMVECs in a dose- and time-dependent manner. (**A**) Effects of PA103 dose and time of infection on inter-endothelial monolayer integrity. (**B**) Effects of PA103 dose and time of infection on PMVEC lysis, measured as LDH release to the culture medium (expressed as % of total LDH). Control PMVECs were inoculated with normal saline solution. (**C**) Time course of LDH release from infected PMVECs comparing control cells, wild type PA103, and the isogenic PA103 (Δ*exoU exoT*::Tc) mutant (bacteria added at MOI = 40:1). (**D**) PA103-mediated lysis of PMVECs (180 min post-inoculation) is dependent on ExoU PLA_2_ activity as demonstrated by addition of the PLA_2_ inhibitor, MAFP (5 µM, added 60 min prior to inoculation and maintained throughout the time course). Data analyzed by one-way ANOVA with Tukey’s post-hoc (**** *p* < 0.0001). (**E**) A comparison of *P. aeruginosa* clinical isolates expressing either STY (JA806, JA808, and JA816) or UTY (JA810, JA811, and JA817) on PMVEC inter-endothelial monolayer integrity. PA103 and an isogenic control (denoted PA103 Δ*exoU exoT*::Tc) lacking exoenzymes are included as a control. (**F**) A comparison of *P. aeruginosa* clinical isolates expressing either STY or UTY on PMVEC lysis, measured as LDH release over time (bacteria added at MOI = 40:1). Data represent n = 3–5 biological replicates.

The overarching goal of this study was to examine the effects of the *P. aeruginosa* T3SS effector, ExoU, on the barrier and inflammatory functions of PMVECs using a cell culture infection model. Our studies here compared two main groups of *P. aeruginosa* strains based on whether or not they express ExoU (Table 1). Strain PA103 is a well-characterized laboratory strain that expresses a functional T3SS along with the ExoU and ExoT effectors, but lacking motility [12]. In addition, we tested isogenic PA103 mutant strains lacking *exoU*, *exoT*, or both. We also tested primary clinical strains isolated from critically ill patients in the ICUs at the University of Alabama at Birmingham [41]. These strains display variable motility profiles (Supplementary Materials, Figure S1), were previously shown to possess a functional T3SS, and either encode ExoU, ExoT, and ExoY or encode ExoS, ExoT, and ExoY [41]. To determine the effects of ExoU on PMVECs, cultured cells were inoculated with the various strains and inter-endothelial gap formation and cell lysis were assessed by microscopy and lactate dehydrogenase (LDH) release, respectively. We first used wild type strain PA103 to establish the assay conditions. The results demonstrate that infection of PMVECs induced cell rounding and inter-endothelial gap formation in a time- and dose-dependent manner (Figure 1A). In addition, PA103 infection induced PMVEC death in a time- and dose-dependent manner as measured by LDH release into the culture medium (Figure 1B). Importantly, LDH release was significantly reduced during infection with an isogenic PA103 strain lacking ExoU and ExoT (Figure 1C) and in the presence of MAFP (a compound that inhibits both ExoU and host-derived PLA_2_ activity, added at 5 µM), indicating that PA103-induced PMVEC death is PLA_2_-dependent (Figure 1D). To further determine whether *P. aeruginosa*-induced PMVEC inter-endothelial gap formation and lysis are driven by ExoU, we tested several clinical isolates. *P. aeruginosa* clinical isolates expressing ExoS, ExoT, and ExoY induced PMVEC rounding/inter-endothelial gap formation (Figure 1E) but did not cause LDH release over the time course tested (Figure 1F). These observations are consistent with previous reports on the effects of ExoS, ExoT, and ExoY in endothelial cells [42,43]. In comparison, *P. aeruginosa* clinical isolates expressing ExoU, ExoT, and ExoY induced PMVEC rounding and inter-endothelial gap formation (Figure 1E), along with time-dependent LDH release (Figure 1F). Together, these data indicate that ExoU is a major determinant of PMVEC barrier disruption and lysis during *P. aeruginosa* infection.

### 2.2. ExoU Triggers Caspase-1 Activation in PMVECs during P. aeruginosa Infection

**Figure 2:**
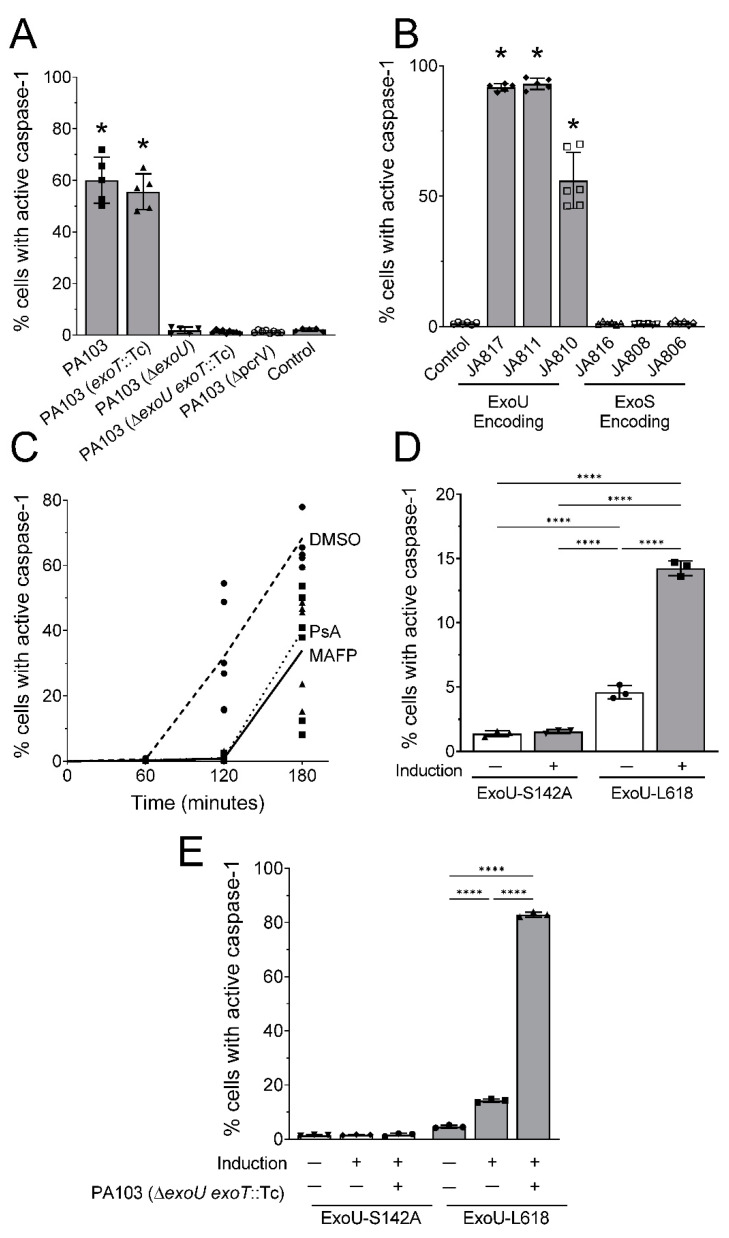
*P. aeruginosa* strains encoding ExoU trigger intracellular caspase-1 activation in PMVECs. (**A**,**B**) *P. aeruginosa* strains expressing ExoU exclusively activate caspase-1 in PMVECs during infection as measured by FLICA and flow cytometry ((**A**) laboratory strain PA103, (**B**) clinical isolates, bacteria inoculated at MOI = 40:1). Data analyzed by one-way ANOVA with Tukey’s post-hoc (* *p* < 0.05). (**C**) Intracellular caspase-1 activation by ExoU depends on PLA_2_ activity as demonstrated by addition of the PLA_2_ inhibitor, MAFP (5 µM), and the ExoU-specific inhibitor Pseudolipasin A (PsA, 50 µM). DMSO was tested as a vehicle control for the PLA_2_ inhibitors. (**D**) ExoU activates intracellular caspase-1 in the absence of infection. PMVECs were engineered to express an inducible ExoU isoform (L618) or an activity-null control (S142A). Caspase-1 activation was measured using the FLICA assay comparing non-induced and induced conditions. Data analyzed by one-way ANOVA with Tukey’s post-hoc (**** *p* < 0.0001). (**E**) Engineered PMVECs were treated as in panel (**D**), except a set of cultures under induction conditions was also inoculated with the PA103 Δ*exoU exoT*::Tc (MOI = 40:1) and caspase-1 activation measured using the FLICA assay. Data analyzed by one-way ANOVA with Tukey’s post-hoc (**** *p* < 0.0001). Data represent n = 3–5 biological replicates.

We have previously determined that PA103 infection of PMVECs elicits activation of the inflammasome-caspase-1 axis [44,45]. Inflammasomes are intracellular pattern recognition receptors whose activation triggers inflammation via pro-caspase-1 autoproteolysis, subsequent processing of pro-IL-1β and pro-IL-18, and Gasdermin D activation to execute a rapid form of cell death termed pyroptosis [46,47,48,49,50]. We have previously validated the use of a membrane permeable, irreversible Fluorescent Labeled Inhibitor of CAspase-1 (FLICA, FAM-YVAD-FMK), and flow cytometry as a single cell assay to identify PMVECs with activated caspase-1 [45]. In uninfected PMVECs, FLICA is freely diffusible and readily washed out of the cell. Upon caspase-1 activation, FLICA binds irreversibly to caspase-1 and becomes trapped inside of the cell allowing enumeration of activated cells by flow cytometry. Here, we sought to determine whether ExoU was responsible for caspase-1 activation in PMVECs by infecting with a series of isogenic PA103 mutant stains. Intriguingly, PA103-dependent activation of caspase-1 in PMVECs was only observed in strains expressing ExoU (Figure 2A). Furthermore, a mutant strain expressing ExoU, but lacking the T3SS, did not activate caspase-1, indicating a requirement for ExoU injection into the host cell cytoplasm (Figure 2A). Moreover, primary *P. aeruginosa* clinical isolates expressing ExoU, ExoT, and ExoY activated caspase-1, whereas clinical isolates expressing ExoS, ExoT, and ExoY did not activate caspase-1 in PMVECs during *P. aeruginosa* infection (Figure 2B). Next, we tested whether ExoU-dependent activation of caspase-1 required enzymatic activity using MAFP and Pseudolipasin A (PsA), a specific inhibitor of ExoU PLA_2_ activity that does not inhibit host PLA_2_ [51]. The vehicle control (DMSO), MAFP (5 µM), or PsA (50 µM) were added 60 min prior to inoculation and maintained throughout the time course. The data in Figure 2C show that inhibiting ExoU PLA_2_ activity delayed and decreased caspase-1 activation in PMVECs during *P. aeruginosa* infection. These data indicate that ExoU PLA_2_ activity is involved in *P. aeruginosa*-induced caspase-1 activation in PMVECs.

To determine whether ExoU alone, in the absence of infection, was able to activate caspase-1 in PMVECs, we used an ectopic expression system to induce intracellular ExoU expression [52]. This system uses a pseudo-lentivirus to integrate the *exoU* coding sequence into the PMVEC genome under the control of a doxycycline-inducible promoter. In this expression construct, ExoU is also tagged with a protein degradation epitope to tightly regulate protein production via addition of a stabilizing compound (Shield1; for further details on the system, please see reference [52]). Two different *exoU* mutants were introduced into PMVECs. Due to the high toxicity of ExoU, our studies employed a previously described ExoU-L618 mutant harboring a 5 amino acid insertion that diminishes, but does not ablate, PLA_2_ activity, allowing for stable integration of the expression cassette into the genome [53]. In addition, we tested an activity-null ExoU-S142A mutant. The data in Figure 2D show that ectopic expression of ExoU-L618 in PMVECs was able to induce caspase-1 activation in the absence of infection, whereas the activity-null ExoU-S142A mutant did not. Intriguingly, infection of the ExoU-L618-expressing PMVECs with the PA103 mutant lacking ExoU and ExoT triggered robust caspase-1 activation (Figure 2E), indicating that other bacteria-associated factors are also involved in caspase-1 activation during *P. aeruginosa* infection. Together, these data indicate that ExoU is able to activate caspase-1 in PMVECs, in a PLA_2_-dependent manner.

### 2.3. ExoU-Induced Reactive Oxidative Species Signaling Contributes to Caspase-1 Activation in PMVECs during P. aeruginosa Infection

**Figure 3:**
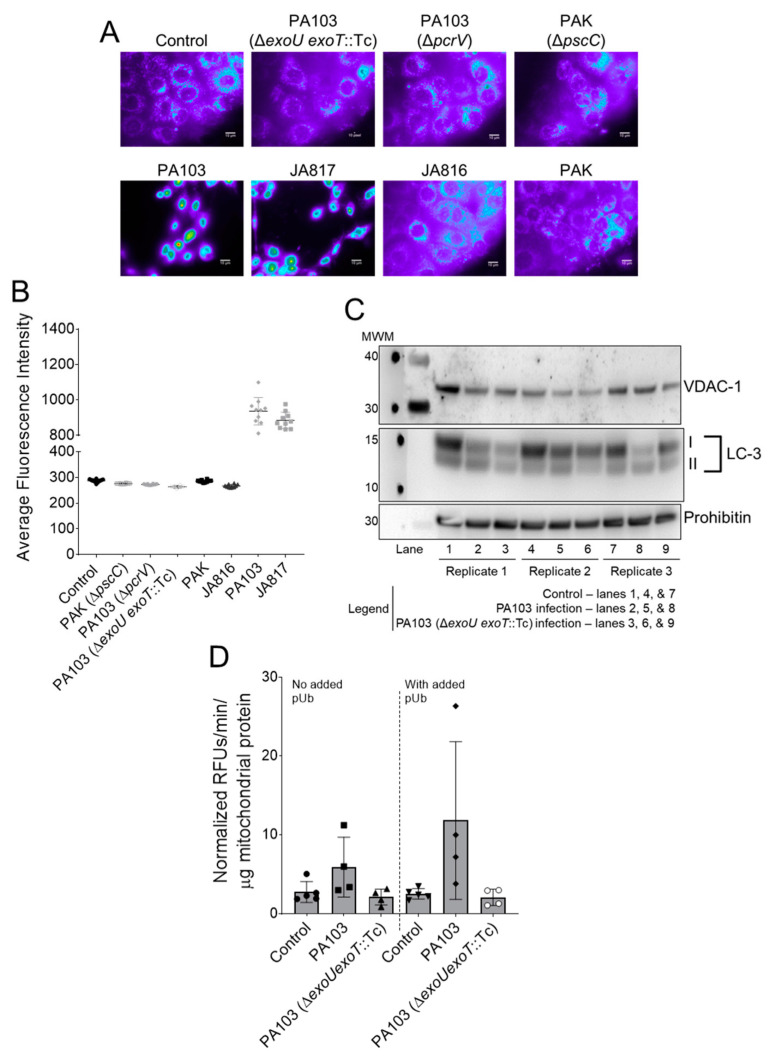
*P. aeruginosa* strains encoding ExoU elicit oxidative stress in PMVECs and ExoU associates with mitochondria. (**A**) PMVECs were loaded with a mitochondria-localized ROS probe, inoculated with the *P. aeruginosa* strains denoted in the figure for 180 min (MOI = 20:1), and images captured (pseudo-colored representative images shown). (**B**) Quantification of ROS probe fluorescence intensity demonstrates that only *P. aeruginosa* strains expressing ExoU elicit high levels of ROS during PMVEC infection. (**C**) Western blot of enriched Mito-MAM fractions probed for the mitochondria-specific proteins VDAC-1 and Prohibitin, and the LC3 marker of autophagic activation. (**D**) ExoU associates with mitochondria during PMVEC infection as indicated by ExoU detection in enriched Mito-MAM fractions using a highly sensitive PLA_2_ assay. ExoU PLA_2_ activity is dependent on addition of its co-factor, poly-Ubiquitin (pUb). Data analyzed by one-way ANOVA with Tukey’s post-hoc (in reactions containing added pUb, *p* < 0.05 when comparing Control to wild type PA103 and when comparing wild type PA103 to PA103 Δ*exoU exoT*::Tc). Data represent n = 3–5 biological replicates.

Thus far, the data suggest ExoU induces an intracellular signal in PMVECs that elicits caspase-1 activation. We postulated that ExoU PLA_2_ activity might damage intracellular organelles, which is a well-established mechanism that triggers caspase-1 activation [54]. Prior evidence from the literature suggests *P. aeruginosa* infection induces mitochondrial oxidative stress and damage [55], but a specific role for ExoU has not been tested. Thus, we determined the effects of ExoU on mitochondrial oxidative stress in PMVECs infected with *P. aeruginosa*. To this end, PMVECs were pre-loaded with MitoSOX indicator, infected with various *P. aeruginosa* strains, and oxidative stress assessed by fluorescent microscopy. Interestingly, infection of PMVECs with *P. aeruginosa* strains expressing ExoU induced a robust fluorescent signal, indicating increased reactive oxygen species production (Figure 3A,B). Conversely, PA103 mutants lacking the T3SS, the PAK laboratory strain, or a clinical isolate expressing ExoS, ExoT, and ExoY, did not increase reactive oxygen species production in infected PMVECs (Figure 3A,B).

The differential effects of ExoU on mitochondrial oxidative stress suggest ExoU might target or associate with mitochondria. To test whether ExoU associates with mitochondria, we first adapted a protocol to enrich mitochondria and mitochondria-associated membrane (Mito-MAM) fractions from control and infected PMVECs. To confirm Mito-MAM enrichment in our model, we verified that extracts contained Prohibitin [56,57] and VDAC-1 [58], two known mitochondria-associated control proteins (Figure 3C). Based on densitometry analyses, the levels of these proteins remained relatively unchanged under control and infection conditions (data not shown). We then assayed for the presence of ExoU in these enriched Mito-MAM fractions using a highly sensitive PLA_2_ enzyme activity assay. It is important to note that this assay is able to distinguish ExoU PLA_2_ activity from host PLA_2_ activity because ExoU is stimulated by its eukaryotic co-factor, poly-ubiquitin (pUb) [14,59]. For these assays, enriched Mito-MAM fractions from control or infected PMVECs were suspended directly into a buffered solution containing a fluorogenic phospholipid substrate (PED6). Each sample was split into two equal reactions (one with and one without added recombinant pUb) and fluorescence measured over a time course. The PED6 hydrolysis rate was determined as background-corrected relative fluorescent units (RFU) normalized to time and to total mitochondrial protein added to each reaction. Intriguingly, pUb-stimulated PLA_2_ activity was only observed in enriched Mito-MAM fractions from PMVECs infected with wild type PA103 expressing ExoU (Figure 3D). The potential localization of ExoU to the mitochondria during infection prompted us to question whether mitochondria were being subjected to damage. To this end, we assayed Mito-MAM fractions for the presence of microtubule-associated protein light chain 3B (LC3) forms LC3-I and LC3-II as canonical markers of autophagy activation [60]. Compared to uninfected controls, there were slightly lower levels of LC3-I and LC3-II in Mito-MAM fractions isolated from infected cells, but overall there was no clear indication that ExoU preferentially triggered autophagy activation at the mitochondria under the conditions tested (Figure 3C). Thus, the detection of ExoU PLA_2_ activity in Mito-MAM fractions implicates an interaction between ExoU and mitochondria during *P. aeruginosa* infection of PMVECs.

**Figure 4:**
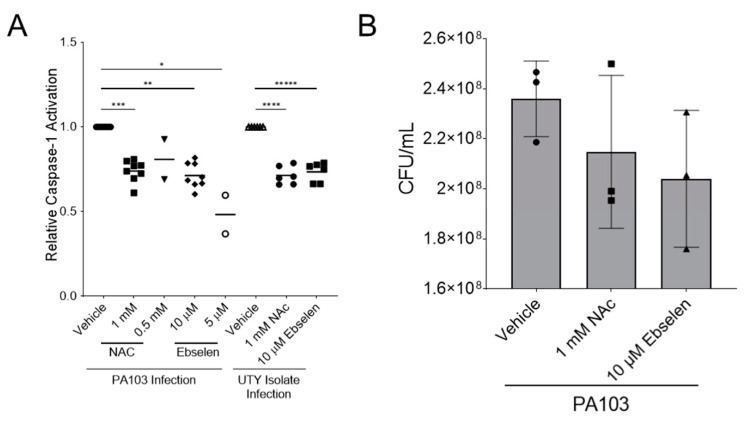
Reactive species scavengers alter ExoU-mediated intracellular caspase-1 activation in *P. aeruginosa*-infected PMVECs. (**A**) Effects of the reactive species scavengers NAC and Ebselen (and their vehicle control) on caspase-1 activation in PMVECs using the FLICA flow cytometry assay. Wild type PA103 (MOI = 40:1) was compared to a clinical isolate expressing UTY (JA817, MOI = 40:1). FLICA signal was analyzed at 180 min post-inoculation. (**B**) A comparison of the effects of NAC and Ebselen on PA103 viability by plate counts for colony forming units (CFU)/mL (n = 3 biological replicates). Data analyzed by one-way ANOVA with Tukey’s post-hoc (* *p* < 0.0001, ** *p* = 0.0003, *** *p* = 0.0019, **** *p* = 0.0040, ***** *p* = 0.0115). Data represent n = 3–5 biological replicates.

Reactive oxygen species are a known danger signal for caspase-1 activation [54,61,62]. Considering that only *P. aeruginosa* strains expressing ExoU trigger caspase-1 activation and reactive oxygen species (ROS) in infected PMVECs, we next tested the effects of various reactive species scavengers on caspase-1 activation. For these experiments, we used N-acetylcysteine (NAC) as a general scavenger of reactive species and Ebselen to scavenge NADPH oxidase-derived species. Each scavenger was first tested at two doses in PA103-infected PMVECs, and then caspase-1 activation was determined using the FLICA assay. Both NAC and Ebselen significantly decreased the levels of caspase-1 activation during PA103 infection (Figure 4A). We also tested the reactive oxygen species scavengers during infection of PMVECs with a primary clinical isolate expressing ExoU (JA817). Similar to the isogenic PA103 strains, the addition of NAC or Ebselen significantly decreased caspase-1 activation levels during JA817 infection (Figure 4A). To verify that the effects of NAC or Ebselen were not trivially due to killing of PA103, we performed plate count assays (Figure 4B). Together, these data suggest that ExoU-induced caspase-1 activation in PMVECs is regulated by the production of reactive oxygen species.

## 3. Discussion

Herein, we present our studies on the role of the *P. aeruginosa* T3SS effector, ExoU, in driving PMVEC barrier disruption and the activation of caspase-1-driven inflammation. Using the *P. aeruginosa* laboratory strain, PA103, PA103 isogenic mutants, and primary clinical isolates from critically ill patients, we showed strains expressing ExoU induce PMVEC barrier disruption and lysis within 3 h post-inoculation. In addition, using a single cell fluorescence-flow cytometry assay, we demonstrated that strains expressing ExoU induce intracellular caspase-1 activation. Importantly, using a combination of genetic and pharmacological approaches, we demonstrated that barrier disruption and caspase-1 activation are dependent on ExoU PLA_2_ enzyme activity. We also showed that infection of PMVECs with ExoU-expressing strains elicited ROS and that ExoU co-associated with mitochondria. Intriguingly, the scavengers NAC and Ebselen ameliorated, in part, ExoU-dependent activation of caspase-1. Together, these data suggest a complex interaction between *P. aeruginosa* and lung endothelial cells that is dependent on ExoU.

Our observation that ExoU causes robust PMVEC barrier disruption and cell lysis (as indicated by LDH release) confirms one of its best-known virulence traits. It is established that, upon injection by the T3SS, ExoU localizes to the host plasmalemmal membrane [12,24,63]. ExoU-membrane association requires C-terminal residues residing between 550–687 and involves binding to phosphatidylinositol 4,5-bisphosphate (PI(4,5)P_2_) on the membrane inner leaflet. ExoU PLA_2_ is subsequently activated through interactions with ubiquitinylated proteins and membrane inositol phosphatides [59,64,65,66,67] and initiates host cell destruction. Using a highly sensitive PLA_2_ enzymatic activity assay, we discovered that *P. aeruginosa* ExoU may also associate with lung endothelial mitochondrial membranes during infection. While we also observed an increase in mitochondrial ROS production, future studies are required to determine the full extent of the ExoU-mediated effects on mitochondrial integrity and function.

In order to establish a lung infection and cause pneumonia, *P. aeruginosa* must survive the initial host innate immune response. ExoU has been shown to play an important role in inhibiting the host innate immune response through interactions with lung-resident alveolar macrophages and epithelial cells, and infiltrating neutrophils [12,23,31,32,33,34,35]. During *P. aeruginosa* infection, ExoU is a potent inducer of rapid cell death that occurs as early as 3 h post-infection [36] and involves the ExoU membrane localization domain [68]. During the early stages of infection, ExoU is injected into lung-resident alveolar macrophages followed by injection into infiltrating neutrophils and monocytes [23]. ExoU-mediated cytotoxicity of resident and infiltrating immune cells facilitates immune avoidance. In addition to inducing epithelial cell death, ExoU also triggers the release of pro-inflammatory mediators such as IL-6, CXCL-8 (IL-8), and arachidonic acid metabolites [34]. Intriguingly, ExoU-mediated production of host-derived pro-inflammatory lipid mediators promotes neutrophil trans-epithelial migration and potentially exacerbates lung injury [33].

The importance of ExoU in disrupting host responses directed by lung endothelial cells is an emerging field of interest [32,36,37,38,39,40]. Our previous work has shown that *P. aeruginosa* infection of PMVECs induces activation of caspase-1-dependent inflammation [44,45]. Intriguingly, the present study strongly suggests ExoU elicits a specific pattern of caspase-1 activation in PMVECs. In innate immune cells such as macrophages, caspase-1 activation typically leads to its release to the extracellular milieu where it can be measured [69]. However, we recently demonstrated that *P. aeruginosa* infection of PMVECs triggers caspase-1 activation that can be measured with an intracellular staining and flow cytometry technique (FLICA) [45]. The data presented herein suggest that ExoU specifically elicits intracellular caspase-1 activation in PMVECs, and the effect is dependent on ExoU PLA_2_ activity. Laboratory and clinical *P. aeruginosa* strains lacking ExoU failed to activate PMVEC intracellular caspase-1. Work is ongoing to determine the specific effects of ExoU-mediated activation of intracellular caspase-1 on PMVEC barrier and inflammatory functions. In addition, our observation that ExoU also preferentially activated ROS generation prompted us to determine whether ROS might also be involved in the intracellular caspase-1 activation. The addition of ROS scavengers (NAC and Ebselen) partially suppressed ExoU-dependent caspase-1 activation. Our observations in PMVECs are consistent with previous studies demonstrating ExoU-mediated ROS imbalances in immune, epithelial, and endothelial cells [36,39,70], which is a key player in driving inflammatory lung injury. Further studies are required to identify whether ExoU targets the Nox2- and Nox4-mediated signaling, which have been previously shown to regulate endothelial cell ROS signaling [71]. An additional question that remains outstanding is whether the ExoU-dependent oxidant source responsible for caspase-1 activation is lipid peroxidation secondary to ExoU PLA_2_ activity. Intriguingly, a recent study determined that ExoU-induced necrosis involves lipid peroxidation [70]. Thus, ExoU-induced lipid peroxidation may trigger multiple effects in PMVECs during *P. aeruginosa* infection leading to host cell death. Finally, future studies, including pre-clinical animal infection models, are required to identify the nature of the inflammasome(s) in PMVECs that respond to ExoU and ROS signaling to elicit intracellular caspase-1 activation along with downstream activation of other proteins, such as apoptotic caspases and cytokines, related to the inflammatory process.

## 4. Conclusions

The *P. aeruginosa* T3SS effector, ExoU, is a potent PLA_2_ that exerts pleiotropic effects on PMVECs during infection. This work demonstrates that *P. aeruginosa* laboratory and primary clinical strains encoding ExoU cause the most damage to PMVECs. In addition, this is the first report that ExoU associates with enriched PMVEC Mito-MAM fractions indicating potential localization to host mitochondria during infection. Furthermore, these data suggest that ExoU-induced oxidative stress in PMVECs is a potential signal to trigger an inflammatory response via activation of caspase-1. Together, these data suggest ExoU induces cytolytic and non-cytolytic effects during infection of PMVECs. Future studies are required to dissect the relative contributions of ExoU-induced reactive oxygen species and caspase-1 activation on PMVEC barrier and inflammatory functions in response to *P. aeruginosa* infection.

## 5. Materials and Methods

### 5.1. Reagents

Pulmonary microvascular endothelial cell (PMVEC) cultures were routinely maintained in Dulbecco’s Modified Eagle Medium (DMEM, Santa Cruz Biotech) supplemented with 10% (vol/vol) of fetal bovine serum (FBS). Cells were dissociated from cell culture dishes using Trypsin-EDTA (0.05%/0.53 mM solution in Hank’s balanced salt solution, without Ca^++^ and Mg^++^, ThermoFisher). For cell culture infections, DMEM without phenol red (ThermoFisher, Waltham, MA, USA) was supplemented with 4 mM L-Glutamine (Corning glutagro supplement) and 1 mM sodium pyruvate (infection medium referred to as scDMEM). Sterile normal saline solution (0.9%) was used for preparation of *P. aeruginosa* for infections. PMVEC damage and lysis was measured using an LDH Cytotoxicity Detection Kit (TakaraClontech, San Jose, CA, USA). PLA_2_ activity was inhibited by addition of MAFP (Methyl arachidonyl fluorophosphonate, MilliporeSigma, Burlington, MA, USA) dissolved in dimethyl sulfoxide (100% anhydrous, DMSO). An ExoU-specific inhibitor, Pseudolipasin A, was a custom synthesis from Chembridge (San Diego, CA, USA) (dissolved in DMSO). Reactive oxygen species were measured using MitoSOX Red (ThermoFisher). PLA_2_ activity was measured in vitro using the fluorogenic PLA_2_ substrate, PED6 (N-((6-(2,4-Dinitrophenyl)amino)hexanoyl)-2-(4,4-Difluoro-5,7-Dimethyl-4-Bora-3a,4a-Diaza-s-Indacene-3-Pentanoyl)-1-Hexadecanoyl-sn-Glycero-3-Phosphoethanolamine, Triethylammonium Salt, ThermoFisher). Caspase-1 activity was measured using a Fluorescent Labeled Inhibitor of CAspase-1 reagent (FLICA, FAM-YVAD-FMK, ImmunoChemstry Technologies, Davis, CA, USA). Scavengers of reactive oxygen species (all from MilliporeSigma) included Mito-TEMPO [(2-(2,2,6,6-Tetramethylpiperidin-1-oxyl-4-ylamino)-2-oxoethyl)triphenylphosphonium chloride], DPI (Diphenyleneiodonium chloride), Ebselen, and NAC (N-acetylcysteine). Western Blots were performed using SDS-PAGE on 4–12% Bis-Tris gels (NuPage, Invitrogen, Carlsbad, CA, USA) in 1X MES-SDS Running buffer (Invitrogen). Molecular weight standards were Novex Sharp Pre-Stained Protein Standard (Invitrogen). Samples were solubilized in Laemmli loading dye (ThermoFisher) containing 2.5% 2-mercaptoethanol (β-ME). Proteins were detected using Mouse monoclonal anti-VDAC1 antibody (Santa Cruz, 1:200 dilution), Rabbit monoclonal anti-LC3A/B antibody (Cell Signaling Technology, 1:1000 dilution), and Rabbit polyclonal anti-Prohibitin antibody (Invitrogen, 1:500 dilution). Secondary antibodies were Goat anti-Mouse-HRP and goat anti-Rabbit-HRP (ThermoFisher, 1:2000 dilution). HRP was detected using Super Signal West Femto (ThermoFisher). Other buffers and reagents used in this study included Lysogeny Broth agar medium (Luria-Bertani, Lennox formulation, ThermoFisher), MOPS (3-(N-morpholino)propanesulfonic acid), sodium chloride (NaCl), TritonX-100 (TX-100), and protease inhibitor cocktail (cOmplete, EDTA-free, MilliporeSigma).

### 5.2. Bacterial Strains and Culture Conditions

All *P. aeruginosa* strains used in this study are described in Table 1. The PA103 derivative *P. aeruginosa* strains used in this study were kindly provided by Dr. Dara Frank (Medical College of Wisconsin). Wild type PA103 encodes ExoU and ExoT. Previous studies have shown individual effector protein mutations in PA103 along with the complemented plasmids restoring the ExoU phenotype [12,72,73]. The PAK derivative *P. aeruginosa* strains used in this study were kindly provided by Dr. Wito Richter (University of South Alabama College of Medicine). Wild type PAK encodes ExoS, ExoT, and ExoY. The *P. aeruginosa* clinical isolates were cultured from patients in the University of Alabama at Birmingham intensive care units and have been previously described [41]. The clinical isolates encode two different combinations of T3SS effectors, namely, ExoU, ExoT, and ExoY or ExoS, ExoT, and ExoY. Motility of the clinical isolates was determined using a soft agar plate assay and measuring the diameter.

Cultures were maintained as frozen stock solutions in nutrient broth supplemented with 12.5% glycerol (at −80 °C). Frozen stocks were transferred to agar plates containing the minimal E salts medium of Vogel and Bonner (VB) and routinely grown overnight at 37 °C. Prior to infection experiments, bacteria were scraped into 10 mL of sterile normal saline solution, collected by centrifugation (5000× *g*, 10 min, at room temperature), and suspended in 1 mL of saline solution. The density of a bacterial suspension was determined by spectrophotometry using the optical density at 600 nm (OD 600_nm_). We experimentally determined the OD 600_nm_ values per colony forming unit (CFU)/mL for each bacterial strain by serial dilution and direct plate counts on Lysogeny Broth agar medium (Luria–Bertani, Lennox formulation, grown overnight at 37 °C) [74].

### 5.3. Eukaryotic Cell Culture and Infection Conditions

Pulmonary microvascular endothelial cells (PMVECs) were of rat origin and isolated from distal lung parenchyma. Isolation and characterization of these cells has been previously described and carried out by the University of South Alabama Center for Lung Biology cell culture core facility. PMVEC cultures were routinely maintained on 100 or 150 mm cell culture dishes in DMEM with FBS, at 37 °C, 5% CO_2_ (humidified environment). Cells were maintained at 80–90% confluence and used between passages 15–20 before a new culture was started from a frozen stock.

PMVEC culture and infection conditions were previously described [45]. Briefly, PMVECs were harvested one day prior to infection by treatment with Trypsin-EDTA (4 min at 37 °C, 5% CO_2_) and washed by centrifugation (500× *g*, 4 min, ambient temperature) into DMEM FBS. Cells were enumerated on a Fuchs-Rosenthal counting chamber, seeded at a density of 5.5 × 10^6^ cell (1 mL DMEM FBS) in 12-well CellBIND-treated culture dishes, and incubated overnight at 37 °C, 5% CO_2_. Approximately 16–18 h later, two wells from the 12-well dish were harvested for counting in order to calculate the multiplicity of infection (MOI). The media on the remaining wells were exchanged by washing with 1 mL of scDMEM (no FBS, phenol-red free), followed by addition of 1 mL of scDMEM (no FBS) and incubation for 1 h at 37 °C, 5% CO_2_. Bacterial suspensions in saline were prepared during the 1 h incubation period as a concentrated stock solution based on the desired final MOI for the experiment. Bacteria stocks were ultimately diluted into scDMEM containing any required additional components (e.g., FLICA reagent, inhibitors, and/or vehicle controls), and a final volume of 0.3 mL was used per culture well. MOIs are reported on the figure or in the figure legends, and control wells were inoculated with scDMEM-containing saline. Plates were incubated at 37 °C, 5% CO_2_, and culture wells analyzed at 60, 120, or 180 min post-inoculation as described in the figure legends.

### 5.4. Cell Damage and Lysis Measurements

Monolayers were imaged for evidence of damage and interendothelial gap formation using a Nikon Eclipse TS100 inverted microscope at 10X magnification. Lactate dehydrogenase (LDH) release from cells was measured as an indicator of cell lysis. Culture supernatants and in whole cell lysates were collected a processed separately for LDH measurements and represented in figures as (Supernatant LDH / (Supernatant LDH + Whole Cell Lysate LDH)) × 100%. Supernatants were collected from duplicate wells and transferred into 1.5 mL microcentrifuge tubes, followed by centrifugation (21,130× *g*, 5 min, room temperature) to remove bacteria. Supernatants were transferred to clean 1.5 mL microcentrifuge tubes. Cells were lysed and scraped into 400 μL of 0.1% TX-100 using a 1 mL micropipette and transferred to individual 1.5 mL microcentrifuge tubes. LDH activity measurements were performed on whole cell lysates diluted 1:10 in 0.1% TX-100. LDH activity measurements were performed on culture medium supernatants either directly or after a 1:10 dilution into scDMEM. Samples were analyzed either immediately or stored at 4 °C for no longer than 2 days prior to assay. LDH catalyst was dissolved as per the manufacturer’s directions. A 0.1 mL volume of each experimental sample was aliquoted in duplicate into 96-well plate and gently mixed with 0.1 mL of LDH reagent. Plates were protected from light and incubated for 30 min at room temperature. Plates were read on a BioRad plate reader spectrophotometer at 490 nm.

### 5.5. FLICA and Flow Cytometry

Determination of PMVEC intracellular caspase-1 activation using the FLICA assay was performed as previously reported [45]. Our previous work describes the optimization of assay conditions and validation of FLICA specificity using a PMVEC line where *caspase-1* was mutated by CRISPR-Cas9 gene engineering [45]. Briefly, culture medium was removed from FLICA-treated PMVECs and any floating cell collected by centrifugation. Adherent cells were gently washed in 1 mL of Apoptosis wash buffer (as per the manufacturer’s instructions). The wash buffer was removed and replaced with 1 mL of fresh wash buffer. Cells were gently scraped, triturated into a single cell suspension, and collected by centrifugation (any cells collected from the culture medium were added back). Cells were then washed two additional times by centrifugation in 1 mL of wash buffer. Cells were subsequently suspended in 0.75 mL of wash buffer and transferred to 5 mL flow cytometry tubes containing 0.25 mL 10% formalin. Fixed cell suspensions were either analyzed immediately or stored at 4 °C protected from light. Analyses were performed on a BD FACS Canto II Flow Cytometry System using the FITC laser and data analyzed using TreeStar’s FlowJo software v10 (FlowJo, LLC, Ashland, OR, USA) as previously described [45].

### 5.6. Reactive Oxygen Species Measurements

PMVECs suspended in DMEM FBS were seeded at 1 × 10^6^ in a 35 mm petri dish with inset coverslip (MatTek, Part No: P35G-1.5-14-C) and grown overnight at 37 °C, 5% CO_2_. The following morning, culture medium was exchanged to 2 mL scDMEM containing 5 µM MitoSOX Red and PMVECs incubated for 1 h at 37 °C, 5% CO_2_. Cultures were subsequently treated with saline (Ctrl) or infected with various *P. aeruginosa* strains for 3 h in scDMEM (MOI of 20). Infection medium was removed and fresh scDMEM added to cells. PMVECs incubated with scDMEM alone served as the negative control. Images were then acquired using a Nikon Eclipse TE2000-U fluorescence microscope (60X objective). Images were analyzed using Metamorph software to determine MitoSOX Red signal intensities. *P. aeruginosa* infection images are representative of three independent experiments performed in duplicate. For each experiment, the average intensity per cell for 10 images across each dish for each experiment was determined. Compiled average intensities per cell are shown in the figure.

### 5.7. Isolation and Assay of Mitochondria and Mitochondria-Associated Membrane Fractions

PMVECs were seeded in a 150 mm petri dish and grown at 37 °C, 5% CO_2_ until confluence (~3.0 × 10^7^ total cells per dish). Subsequently, cells were washed into scDMEM and incubated at 37 °C, 5% CO_2_ for 1 h prior to inoculation. Culture medium was replaced with 10 mL scDMEM containing saline (Ctrl), or PA103 derivative strains at a MOI of 40, followed by incubation at 37 °C, 5% CO_2_ for 3 h. Subsequently, culture medium was removed, and any floating cells were collected by a low-speed centrifugation first (500× *g*, 4 min, room temperature). Adherent cells were harvested by dissociation in Trypsin-EDTA, collected by centrifugation, and combined with the cells collected from the culture medium. Cell pellets were suspended in final volume 1 mL of DMEM + 10% FBS then transferred to a 2 mL microfuge tube. Mitochondria and mitochondria-associated membrane (Mito-MAM) fractions were then enriched using Mitochondria Isolation Kit for Cultured Cells as per the manufacturer’s instructions (ThermoFisher). To assess the efficacy of mito-MAM fraction enrichment, we measured levels of Prohibitin, a known mitochondria-associated control protein [56,57], in a Western blot. As a secondary marker, we also measured VDAC-1, which is known to increase and form mitochondrial outer membrane pores under mitochondrial stress [58]. It is important to note that we also detected varying levels of the cytosolic proteins β-actin and glyceraldehyde phosphate dehydrogenase (GAPDH) in the mito-MAM fractions by Western blot (data not shown). Thus, we refer to the mito-MAM fractions as enriched to acknowledge possible contributions from other cytosolic membrane fractions.

To assay for the presence of ExoU, enriched mito-MAM fractions were directly suspended in 50 mM MOPS (pH 7.4)/ 50 mM NaCl buffer solution containing the fluorogenic PLA_2_ substrate, PED6 (N-((6-(2,4-Dinitrophenyl)amino)hexanoyl)-2-(4,4-Difluoro-5,7-Dimethyl-4-Bora-3a,4a-Diaza-s-Indacene-3-Pentanoyl)-1-Hexadecanoyl-sn-Glycero-3-Phosphoethanolamine, Triethylammonium Salt, 29.7 µM) [75]. The PED6 assays were conducted as previously described [17,41,45]. Briefly, reactions were pre-incubated (ambient temperature) for 25 min to facilitate formation of enzyme–substrate complexes followed by a measurement of the baseline fluorescence signal. Then, poly-ubiquitin (pUb, 0.1 mg/mL) was added to activate ExoU. A separate set of negative control reactions were set up without pUb addition. Fluorescence was then measured every hour for 3 h using NanoDrop 3300 Microvolume Fluorospectrometer. Excitation—470 nm ± 10; emission—511 nm; fluorometer scan—500–750 nm. Data points were corrected by subtracting fluorescence intensity measured in the background reaction incubated with substrate (expressed as relative fluorescent units, RFU). The rate of PED6 hydrolysis was expressed as RFUs/min and normalized to the total mitochondrial protein added to the reaction.

### 5.8. Statistical Analyses of Data

Data are reported as mean ± standard error from at least three independent experiments. Prism 8 (GraphPad Software, San Diego, CA, USA) was used for all analyses. The Shapiro–Wilk test was used to determine normality [76]. For multiple comparison analysis, two-way ANOVA followed by Tukey’s post-hoc test was performed. Differences with a *p* value < 0.05 were considered significant (*p* values are reported in the figure legends).

## Figures and Tables

**Table 1:** Bacterial strains used in this study.

Bacterial Strain	Genotype	Phenotype	Source
PA103	Wild type	Virulent expressing functional T3SS, ExoU, ExoT	D. W. Frank
PA103ΔU	Δ*exoU*	Attenuated virulent expressing functional T3SS, ExoT	D. W. Frank
PA103ΔT	*exoT*::Tc	Attenuated virulent expressing functional T3SS, ExoU	D. W. Frank
PA103ΔUT	Δ*exoU exoT*::Tc	Attenuated virulent expressing functional T3SS	D. W. Frank
PA103Δ*pcrV*	Δ*pcrV*	Avirulent lacking T3SS, expressing ExoU, ExoT	D. W. Frank
JA806, JA808, JA816	Wild type	Clinical isolate expressing functional T3SS, ExoS, ExoT, ExoY	B. M. Wagener, J-F Pittet, W. Richter
JA810, JA811, JA817	Wild type	Clinical isolate expressing functional T3SS, ExoU, ExoT, ExoY	B. M. Wagener, J-F Pittet, W. Richter
PAK	Wild type	Virulent expressing functional T3SS, ExoS, ExoT, ExoY	W. Richter
PAKΔ*pscC*	Δ*pscC*	Avirulent lacking T3SS, expressing ExoS, ExoT, ExoY	W. Richter

## Data Availability

Not applicable.

## Supplementary Materials

The following supporting information can be downloaded at: https://www.mdpi.com/article/10.3390/toxins14020152/s1, Figure S1: Motility assay on the *P. aeruginosa* clinical isolates used in these studies.
