# Supplementary material for: ExoU Induces Lung Endothelial Cell Damage and Activates Pro-Inflammatory Caspase-1 during Pseudomonas aeruginosa Infection
Source: Toxins (Basel). 2022 Feb 18;14(2):152. doi: 10.3390/toxins14020152 (PMC8878379; doi:10.3390/toxins14020152)
Supplement: Supplementary file 1 [file toxins-14-00152-s001.zip › toxins-1576057-supplementary.pdf]

toxins

MDPI

Supplementary Material

ExoU Induces Lung Endothelial Cell Damage and Activates Pro-Inflammatory Caspase-1 during *Pseudomonas aeruginosa* Infection

Kierra S. Hardy, Amanda N. Tuckey, Phoibe Renema, Mita Patel, Abu-Bakr Al-Mehdi, Domenico Spadafora, Cody A. Schlumpf, Robert A. Barrington, Mikhail F. Alexeyev, Troy Stevens, Jean-Francois Pittet, Brant M. Wagener, Jon D. Simmons, Diego F. Alvarez and Jonathon P. Audia

Supplemental Figure 1.

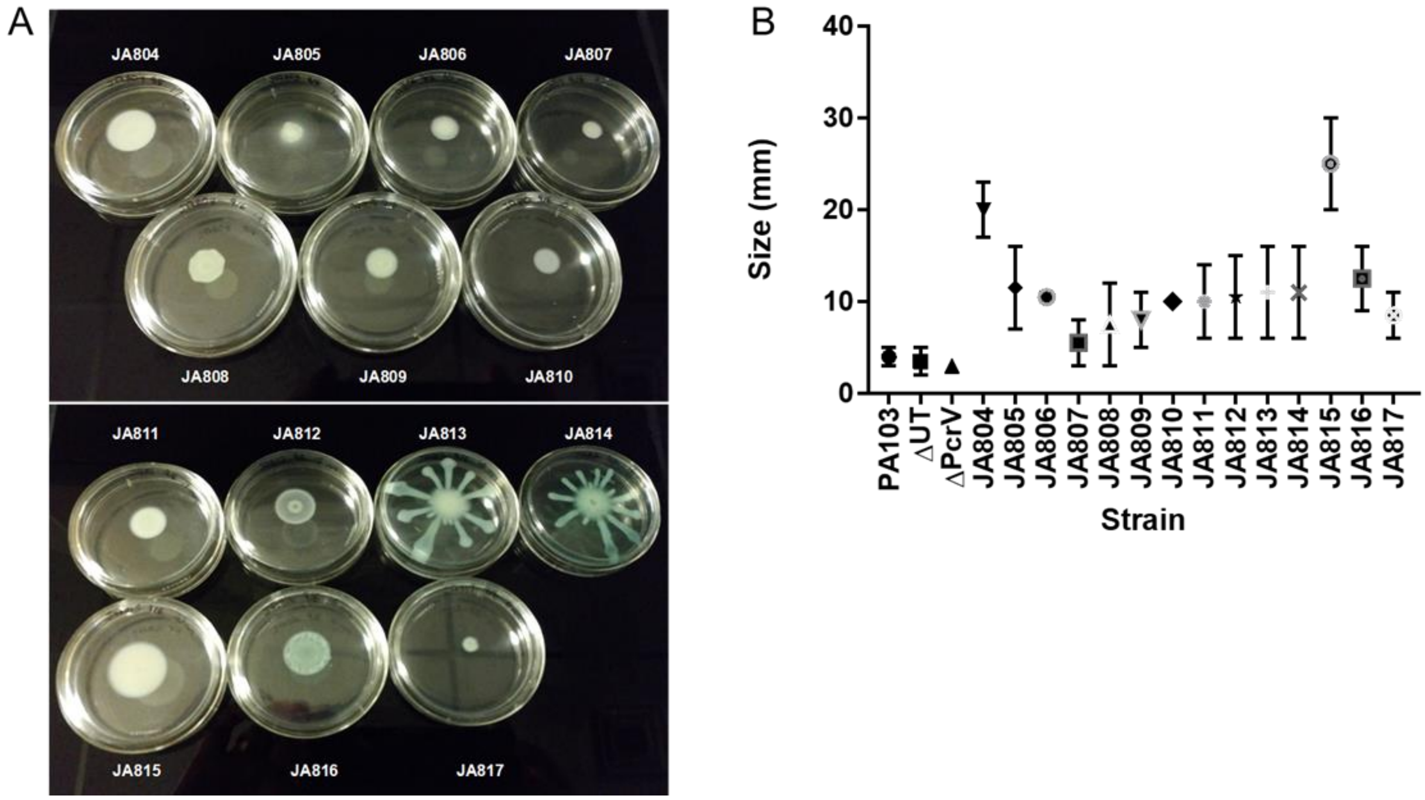

Figure S1. Motility assay on the *P. aeruginosa* clinical isolates used in these studies. (**A**) Images of motility agar plates (representative image). (**B**) Quantification of the motility data (n = 3 biological replicates).
